# Measles in adults: A comparison of hospitalised HIV-infected and HIV-uninfected patients

**DOI:** 10.4102/sajhivmed.v20i1.877

**Published:** 2019-08-13

**Authors:** Nina E. Diana, Charles Feldman

**Affiliations:** 1 Division of Nephrology, Department of Internal Medicine, Charlotte Maxeke Johannesburg Academic Hospital, Johannesburg, South Africa; 2 Division of Pulmonology, Department of Internal Medicine, Charlotte Maxeke Johannesburg Academic Hospital, Johannesburg, South Africa; 3 Faculty of Health Sciences, University of the Witwatersrand, Johannesburg, South Africa

**Keywords:** Measles, Adults, Complications, Human immunodeficiency virus, Infectious diseases

## Abstract

**Background:**

Although measles is traditionally a childhood illness, there are an increasing number of adult cases. Despite both measles and HIV infection being endemic in sub-Saharan Africa, there are little data regarding outcomes in co-infected patients.

**Objectives:**

Compare demographic, clinical, laboratory and radiographic features, as well as outcome (length of hospital stay, complications and mortality) between HIV-infected and HIV-uninfected adult patients admitted with confirmed measles.

**Methods:**

We conducted a retrospective record review of adult patients with confirmed measles who were admitted to the Infectious Diseases Unit at the Charlotte Maxeke Johannesburg Academic Hospital during the peak of the 2009 and 2010 South African measles outbreak. The data collected included demographic, clinical and laboratory parameters, as well as outcomes.

**Results:**

Of the 33 confirmed measles cases admitted, 24 patients were tested for HIV infection and 18 tested seropositive. There were no significant differences in the demographics, clinical findings or laboratory data when comparing the HIV-positive and HIV-negative cases. Serious clinical manifestations were seen more frequently in HIV-positive patients (odds ratio [OR] 5, 95% confidence interval [CI] 0.48–51.8, *p* = 0.34). One of the six patients testing HIV-negative developed pneumonia, while six of the 18 HIV-positive patients had a course complicated by pneumonia. Five of these HIV-positive patients required admission to the intensive care unit, three developing respiratory failure necessitating mechanical ventilation. HIV-positive patients had several other manifestations, including acute kidney injury, purulent conjunctivitis, pancreatitis and encephalitis. HIV-positive patients had a significantly longer hospital stay (*p* = 0.03). There were three deaths in the HIV-positive group, but none in the HIV-negative group (OR 2.9, 95% CI 0.13–65.3, *p* = 0.55).

**Conclusion:**

Our study provides data on the largest series of hospitalised adults infected with HIV and co-infected with measles. More severe consequences seemed to occur in hospitalised HIV-positive patients.

## Introduction

Measles is one of the most contagious of all human viruses,^1^ and infects approximately 10 million people annually, with an estimated mortality of 134 200 in 2015, occurring mainly in developing countries.^2^ Although it is historically a childhood illness,^1^ reports have highlighted an increasing frequency of measles occurring in young adults.^3,4,5,6^ With the incorporation of measles vaccination into routine childhood vaccination programmes, there has been a ‘shift of disease burden’. It now occurs more commonly in older patients who missed vaccination as children.^7,8^ In addition, protection induced by the measles vaccine also seems to wane over a period of years (secondary vaccine failure), with the length of protection having been estimated to be approximately 25 years.^9^

Factors affirming the theory that the HIV epidemic may enhance dissemination of measles include the high rates of primary and secondary measles vaccine failure in HIV-infected persons,^10,11,12,13,14^ atypical presentations of measles resulting in delayed diagnosis in HIV-infected individuals,^15,16^ as well as the association of HIV infection with prolonged measles viral shedding and delayed clearance of the measles virus (MV).^17^ Serious complications have been described in HIV-positive patients co-infected with measles.^18,19,20,21,22,23^

All studies comparing HIV-positive and HIV-negative patients infected with measles have been conducted in the paediatric population only. Moss et al.^24^ reported HIV-positive children to have a longer duration of illness (*p* = 0.03), a longer hospital stay (*p* = 0.0004) and a higher mortality (*p* < 0.01). However, in a report by Sension et al.^25^ from Kinshasa, there were similar rates of pneumonia, diarrhoea and death in the HIV-positive and HIV-negative children. In South Africa, there have been three studies comparing HIV-positive and HIV-negative children infected with measles. Morrow et al.^26^ concluded that although HIV-infected patients were 1.6 times more likely to be hospitalised, there was no difference in death rate between the two groups. Le Roux et al.^27^ documented that the length of hospital admission was longer, the number of re-admissions was greater and the odds ratio (OR) of death was seven times higher in the HIV-positive group. Pamacheche et al.^28^ described the clinical profile of children admitted with measles to a teaching hospital in Johannesburg. There were two deaths, both in children that were HIV-negative.

Between 2009 and 2011, an outbreak of measles occurred in South Africa that resulted in 18 431 laboratory-confirmed cases being reported to the National Institute of Communicable Diseases of the National Health Laboratory Service.^29^ During this outbreak, a number of cases were admitted to the adult Infectious Diseases ward at the Charlotte Maxeke Johannesburg Academic Hospital (CMJAH) in Johannesburg and this afforded us the opportunity to describe the clinical features and outcome of adult patients with measles comparing the HIV-positive and HIV-negative cases.

## Methods

This was a retrospective record review of adult patients with confirmed measles, who were admitted to the Infectious Diseases Unit (IDU) at the CMJAH during the peak of the 2009 and 2011 South African measles outbreak. The majority of cases occurred from week 37 in 2009 to week 24 in 2010. The study period was 29 September 2009 to 31 March 2010.

The data collected included all available demographic and clinical features and laboratory parameters. Patients with clinical features suggestive of measles were confirmed to have measles by serological testing, using the Enzygnost Anti-Measles Virus/IgM assay (Dade-Behring, Marburg, Germany). HIV testing, using a chemiluminescent microparticle immunoassay for the simultaneous qualitative detection of HIV p24 antigen and antibodies to HIV type 1 and/ or type 2 (ARCHITECT HIVAb/Ag Combo Calibrator, Abbott Laboratories, Wiesbaden, Germany), was offered to all confirmed measles cases. The clinical characteristics and outcomes of these patients were compared in HIV-positive and HIV-negative cases. Outcome measures were length of hospital stay, complications and mortality.

The HIV-infected and HIV-uninfected groups were compared using the Mann–Whitney *U* test for continuous variables, and the Fisher’s exact (two-tailed) test for categorical variables. Analyses were done using GraphPad InStat version 3. A *p*-value < 0.05 was considered to be statistically significant.

## Ethical consideration

Permission to conduct the study was obtained from the Human Research Ethics Committee of the University of Witwatersrand (Clearance Certificate No. M10104).

## Results

**FIGURE 1:**
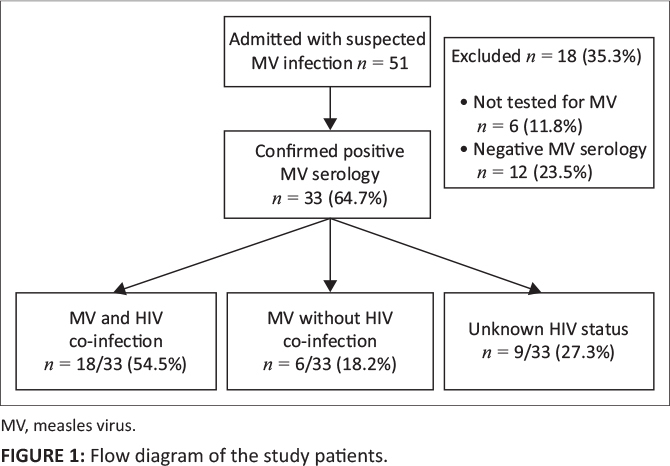
Flow diagram of the study patients.

A total of 51 adult patients with suspected measles were admitted to the IDU of CMJAH between 29 September 2009 and 31 March 2010. Thirty-three (64.7%) of these patients were confirmed to have measles by serology. In 12 patients (23.5%), measles serology was negative and six patients (11.8%) were not tested. Of the 33 patients confirmed to have measles by serology, 24 (72.7%) consented to a test for HIV infection. These 24 patients were studied further. Figure 1 shows the study population included in this analysis.

Of the 24 patients, 13 were female and 11 were male. The mean (standard deviation) age of the HIV-positive group was 28.1 (5.6) years and of the HIV-negative group was 29.6 (9.0) years. Of the 24 patients, 18 patients (12 females) tested seropositive for HIV infection. Of the entire group, only one HIV-positive patient reported a measles contact and two patients in the HIV-positive cohort remembered previously being vaccinated against measles as a child.

Presenting features and examination findings are depicted in Tables 1 and 2. There were no significant differences between the two patient groups. The median duration of symptoms was 4 days (range: 1–7 days) in the HIV-positive group and 3 days (range: 3–6 days) in the HIV-negative group.

**TABLE 1:** Presenting symptoms in 24 adult patients with measles: Comparison of HIV-positive and HIV-negative cases.

Presenting symptom	HIV-positive patients			HIV-negative patients			*p*
	*n*	*N* †	%	*n*	*N* †	%	
Fever	17	18	94.4	6	6	100	1.0
Rash	18	18	100	6	6	100	1.0
Red eyes or conjunctivitis	18	18	100	6	6	100	1.0
Coryza	9	18	50	3	6	50	1.0
Cough	11	18	61.1	6	6	100	0.13
Shortness of breath	2	18	11.1	0	6	0	1.0
Vomiting	3	16	18.8	3	4	75	0.06
Diarrhoea	2	17	11.8	0	4	0	1.0
Abdominal pain	1	17	5.9	0	4	0	1.0
Headache	1	18	5.6	2	6	33.3	0.22

† , *n* denotes the number of patients in whom the symptom was present; *N* is the number of patients in whom the presence or absence of the symptom was recorded.

**TABLE 2A:** Examination findings in 24 adult patients with measles: Comparison of HIV-positive and HIV-negative cases.

Vital signs	Sub-indicator	HIV-positive patients (*N* = 18)				HIV-negative patients (*N* = 6)				*p*-value
		Median	Range	$\bar{x}$	s.d.	Median	Range	$\bar{x}$	s.d.	
Temperature (°C)	-	39	37.5–40	-	-	39	37–39	-	-	0.1
Blood pressure (mmHg)	Systolic	103.5	90–149	-	-	123.5	103–129	-	-	0.13
	Diastolic	69.5	40–99	-	-	75	67–79	-	-	0.42
Heart rate (beats/min)	-	-	-	124	23	-	-	105	23	0.1

**TABLE 2B:** Examination findings in 24 adult patients with measles: Comparison of HIV-positive and HIV-negative cases.

Clinical features	Sub-indicator	HIV-positive patients (*N* = 18)		HIV-negative patients (*N* = 6)		*p*-value
		*n*	%	*n*	%	
Rash	-	18	100	6	100	1.00
Conjunctivitis	-	18	100	6	100	1.00
Koplik spots	-	5	27.8	1	16.7	1.00
Oral thrush	-	6	33.3	0	0	0.28
Lymphadenopathy	-	6	33.3	2	33.3	1.00
Confusion	-	3	16.7	0	0	0.55
Chest examination	Crackles	5	27.8	1	16.7	1.00
	Bronchial breathing	1	5.6	0	0	1.00

Laboratory investigations also revealed no significant differences between the two groups (Table 3). In the HIV-positive group, the median CD4 count was 109 cells/mm³ (range: 18–599 cells/mm³) (16 patients had data), the HIV viral load (VL) was available for seven patients (with a median of 15 000 copies/mL) and three patients had a VL lower than the detectable limit. Eight patients (44.4%) were newly diagnosed with HIV infection on this presentation and four patients were already on highly active antiretroviral therapy.

**TABLE 3:** Laboratory findings in 24 adult patients with measles: Comparison of HIV-positive and HIV-negative cases.

Findings	HIV-positive patients (*n* = 18)				HIV-negative patients (*n* = 6)				*p*-value
	Median	Range	$\bar{\text{x}}$	s.d.	Median	Range	$\bar{\text{x}}$	s.d.	
White cell count (cells x10^9^/l)†	6.6	4–14.8	-	-	6.9	5.8–16	-	-	0.40
Haemoglobin (g/dl)	13.1	9.3–16.3	-	-	16.5	9.6–17.4	-	-	0.11
Platelets (cells x10^9^/l)	-	-	187.7	53.9	-	-	183.0	49.9	0.87
Sodium (mmol/l)	135.5	122–140	-	-	132.5	126–141	-	-	0.24
Potassium (mmol/l)	-	-	4.0	0.6	-	-	4.0	0.6	0.97
Bicarbonate (mmol/l)	-	-	21.7	3.4	-	-	23.8	3.5	0.23
Urea (mmol/l)	-	-	9.6	6.1	-	-	7.2	2.3	0.18
Creatinine (µmol/l)	-	-	117.9	55.2	-	-	100.5	45.7	0.46
Albumin (g/l)	-	-	36.1	6.2†	-	-	34.3	6.7‡	0.65
CRP (mg/l)	-	-	120.8	74.9†	-	-	110.6	58.5¶	0.76

† , number of cases with available data = 17.

‡ , number of cases with available data = 4.

¶ , number of cases with available data = 5.

Serious clinical manifestations were seen more frequently in HIV-positive than HIV-negative patients (OR 5, 95% confidence interval [CI] 0.48–51.8, *p* = 0.34). One of the six patients testing HIV-negative developed pneumonia, while six of the 18 HIV-positive patients had a clinical course complicated by pneumonia (OR 2.5, 95% CI 0.23–26.5, *p* = 0.63). Five of these HIV-positive patients required admission to the intensive care unit or high care unit, three developing respiratory failure necessitating mechanical ventilation. These three patients also developed acute kidney injury. HIV-positive patients had several other manifestations, including purulent conjunctivitis, pancreatitis and encephalitis (Table 4).

**TABLE 4:** Complications in 24 adult patients with measles: Comparison of HIV-positive and HIV-negative cases.

Complication	HIV-positive patients (*N* = 18)		HIV-negative patients (*N* = 6)		*p*-value
	*n*	%	*n*	%	
Any complication	9	50.0	1	16.7	0.34
Pneumonia	6	33.3	1	16.7	0.63
ICU/ high care admission	5	27.8	0	0	0.28
Respiratory failure	3	16.7	0	0	0.55
Mechanical ventilation	3	16.7	0	0	0.55
Acute kidney injury	3	16.7	0	0	0.55
Purulent conjunctivitis	1	5.6	0	0	1
Pancreatitis	1	5.6	0	0	1
Encephalitis	1	5.6	0	0	1

HIV-positive patients had a significantly longer hospital stay (*p* = 0.03). There were three deaths in the HIV-positive group, but none in the HIV-negative group. The OR of death was 2.9 times higher in the HIV-positive group (OR 2.9, 95% CI 0.13–65.3, *p* = 0.55).

## Discussion

In this study, there were no differences in demographic, clinical and laboratory parameters when comparing the HIV-positive and HIV-negative groups. There was a tendency for complications to be more common in the HIV-positive group; however, the only significant difference was a longer length of hospital stay.

Over the 6-month period, 51 adult patients were admitted with suspected MV infection. Despite having clinical features suggestive of MV infection, 12 (23.5%) patients had negative MV serology. Negative serological testing may be attributed to possible laboratory error, to patients not having mounted an adequate immune response because of underlying immunocompromise or to undetectable antibody levels within the first 72 h of the exanthem appearing.^30^

Of the 13 females who consented to HIV serological testing, 12 tested HIV-positive. There were twice as many females as males in the subgroup infected with HIV. This may reflect the burden of HIV infection among women in the South African population.^31^

In 2012, the estimated adult (15–49 years) prevalence rate of HIV and/or AIDS in South Africa was 18.8%.^31^ However, in our study the prevalence of HIV-positivity was 18 of 24 patients (75%). This higher rate of HIV infection among our measles cases may be the result of HIV-infected patients being at increased risk of acquiring measles and requiring hospitalisation^26^; firstly because HIV-induced immune deficiencies are compounded with the immune-suppressive effect of the MV and secondly because of an inferior response to measles vaccination.^10,11,32,33,34,35^

Presenting symptoms, findings on clinical examination and laboratory results revealed no significant differences between the HIV-infected and HIV-uninfected subgroups. All of the patients infected with HIV presented with features typical of MV infection, including the occurrence of a morbilliform rash. This contrasts with published data documenting atypical findings in HIV-infected patients.^15,16^ Furthermore, this is also despite the median CD4 cell count of 109 cells/mm³ in the HIV-infected subgroup, suggesting advanced retroviral disease and immunosuppression.

Measles is typically a self-limiting illness, but individuals who are immunocompromised are at increased risk of severe disease.^24^ This was mirrored in our study as half of the HIV-infected adults in our cohort developed complications related to MV infection, as compared to only one patient in the HIV-uninfected subgroup (OR = 5, 95% CI 0.48–51.8, *p* = 0.34). The length of hospital stay was significantly higher in the HIV-infected subgroup (*p* = 0.03). All three deaths recorded in our cohort occurred in the HIV-infected subgroup (OR = 2.9, 95% CI 0.13–65.3, *p* = 0.55), resulting in a case fatality rate of 16.7% in this group.

Possible limitations of this study include the following. Firstly, there were small patient numbers and this may have limited our ability to show statistical significance in some of the endpoints. Secondly, it was a retrospective study and so the datasets were not complete. Thirdly, we included only patients confirmed to have measles on serological testing, thus excluding 12 cases with clinical features of measles that tested negative for measles by serology and which may have represented false-negative cases because of HIV co-infection with deficient antibody synthesis. Fourthly, only patients with more severe disease were included as they were hospitalised patients. Lastly, the cases were all from a single centre, and therefore the results may not be generalisable. However, our study provides data on the largest series of hospitalised adults infected with HIV and co-infected with measles. Unlike other published literature, we were also able to provide a comparison of adult patients infected and uninfected with HIV, within the same cohort.

## Conclusion

Our findings confirm that MV is still an important cause of morbidity and mortality, even among adult patients. Co-infection with HIV may be associated with worse outcomes. Future studies with larger patient numbers may substantiate this conclusion. HIV testing should be carried out in all adults with suspected MV infection. ‘Mop-up’ vaccination campaigns should perhaps also target adults infected with HIV with the aim of attaining protective antibody levels and reducing the risk of developing disease.
